# Retraction: Pigment epithelium-derived factor promotes the growth and migration of human esophageal squamous cell carcinoma

**DOI:** 10.3389/fonc.2026.1811216

**Published:** 2026-02-24

**Authors:** Frontiers Editorial Office

Following publication, concerns were raised regarding the integrity of the images in the published figures.

During the investigation, the authors failed to provide a satisfactory explanation during the investigation, which was conducted in accordance with Frontiers’ policies. As a result, the data and conclusions of the article have been deemed unreliable and the article has been retracted.

This retraction was approved by the Chief Editors of Frontiers in Oncology and the Chief Executive Editor of Frontiers. The authors agree to this retraction.

